# Viral fibrotic scoring and drug screen based on MAPK activity uncovers EGFR as a key regulator of COVID-19 fibrosis

**DOI:** 10.1038/s41598-021-90701-w

**Published:** 2021-05-27

**Authors:** Elmira R. Vagapova, Timofey D. Lebedev, Vladimir S. Prassolov

**Affiliations:** 1 Engelhardt Institute of Molecular Biology, Russian Academy of Sciences, Moscow, 119991 Russia; 2 Center for Precision Genome Editing and Genetic Technologies for Biomedicine, Engelhardt Institute of Molecular Biology, Russian Academy of Sciences, Moscow, Russia

**Keywords:** Drug discovery, Imaging, Data mining, Data processing, Machine learning, Virtual drug screening, Cell signalling

## Abstract

Understanding the molecular basis of fibrosis, the lethal complication of COVID-19, is urgent. By the analysis of RNA-sequencing data of SARS-CoV-2-infected cells combined with data mining we identified genes involved in COVID-19 progression. To characterize their implication in the fibrosis development we established a correlation matrix based on the transcriptomic data of patients with idiopathic pulmonary fibrosis. With this method, we have identified a cluster of genes responsible for SARS-CoV-2-fibrosis including its entry receptor ACE2 and epidermal growth factor EGF. Then, we developed Vi-Fi scoring—a novel drug repurposing approach and simultaneously quantified antiviral and antifibrotic activities of the drugs based on their transcriptomic signatures. We revealed the strong dual antifibrotic and antiviral activity of EGFR/ErbB inhibitors. Before the in vitro validation, we have clustered 277 cell lines and revealed distinct COVID-19 transcriptomic signatures of the cells with similar phenotypes that defines their suitability for COVID-19 research. By ERK activity monitoring in living lung cells, we show that the drugs with predicted antifibrotic activity downregulate ERK in the host lung cells. Overall, our study provides novel insights on SARS-CoV-2 dependence on EGFR/ERK signaling and demonstrates the utility of EGFR/ErbB inhibitors for COVID-19 treatment.

## Introduction

Severe acute respiratory syndrome coronavirus 2 (SARS-CoV-2) is the cause of the ongoing coronavirus disease (COVID-19) pandemic. Opposed to the majority of coronaviruses, SARS-CoV-2 replicates in the lower respiratory tract resulting in the development of pneumonia, which progresses into acute respiratory distress syndrome (ARDS) with fatal destruction of the human organism in severe cases^1,2^. The general step in ARDS development is the elevation of plasma pro-inflammatory cytokines and rapid lung infiltration with immune cells^3^. In the advanced stages of COVID-19 pulmonary fibrosis and cytokine release syndrome (CRS) is often present^4^. Vast release of proinflammatory cytokines along with sepsis and massive multiorgan damage is the cause of at least 30% of fatal COVID-19 cases^1^*.* Molecular pathways that are responsible for the development of SARS-CoV-2 induced fibrosis, ARDS and CRS, are not yet understood. This gap in the knowledge limits the development of novel therapies targeting the development of fibrosis or the repurposing of existing medicines that may be effective against SARS-CoV-2 infection and virus-induced fibrosis.

It is known that viruses could interfere with pro-survival pathways of the host cell. Some viruses are known to hijack ERK activity in host cells to regulate virus transcription^5^. MAPK/ERK pathway is tightly implemented in cancer cell survival and therapy-resistance through the hyperactivation of cytokine signaling^6^. However, it is not clear whether drugs targeting those pathways are beneficial for the treatment of viral infections or fibrosis.

Recent reports reveal that more than 50% of COVID-19 patients with lung, blood, and esophageal cancer are highly suspected to develop critical symptoms of COVID-19 resulting in a high rate of ICU admission (more than 20%) and high mortality rates compared to other cancers^7^. Immunocompromised patients with several diseases, such as hypertension, chronic obstructive disease (COPD), diabetes, and cardiovascular disease, are more susceptible to the disease caused by SARS-CoV-2 and its associated complications^8,9^. Advanced stages of COVID-19 progression resemble features observed in COPD, idiopathic pulmonary fibrosis (IPF), and lung cancer. All mentioned diseases are tightly linked with the recruitment and suppression of immune cells along with necroptosis or pyroptosis of lung cells^10^ resulting in widespread organ inflammation and CRS, but still, the factors that trigger these processes in individuals are not completely understood. Possibly the heterogeneity in cytokine-involved response could depend on the host cell transcriptomic predisposition, and the expression of *ACE2* and *TMPRSS2* in lung tissues^11^.

So far, the anti-COVID-19 effective therapy is not developed, along with the absence of risk stratification for cancer patients based on their ongoing treatment. Recently developed drug screening and repurposing strategies aim at the revealing of virus or host targeting drugs have several limitations: they are either restricted to BSL3 laboratories or limited to in silico effectiveness quantification^12^.

For the first time, we present our Vi-Fi algorithm for drug repurposing that quantifies both antiviral and anti-inflammatory actions of any drug based on its transcriptomic signature. We combined it with our safe and flexible lentiviral-based system for in vitro verification^13^. As a result, we identified 19 drugs with promising dual (antifibrotic and antiviral) potential, the majority of which are EGFR/ErbB inhibitors, and showed their efficacy in vitro. We also, measured the ERK activity in single lung cells, and show that the drugs with the predicted antifibrotic activity downregulate ERK in lung cells and thus ERK quantification can be used for drug screening. We believe that our findings and novel approaches can be beneficial not only for the development of an effective SARS-CoV-2 treatment strategy but also can be easily translated into other research areas.

## Results

### SARS-CoV-2 induced cytokine signature defines types of lung cancer cell lines

**Figure 1:**
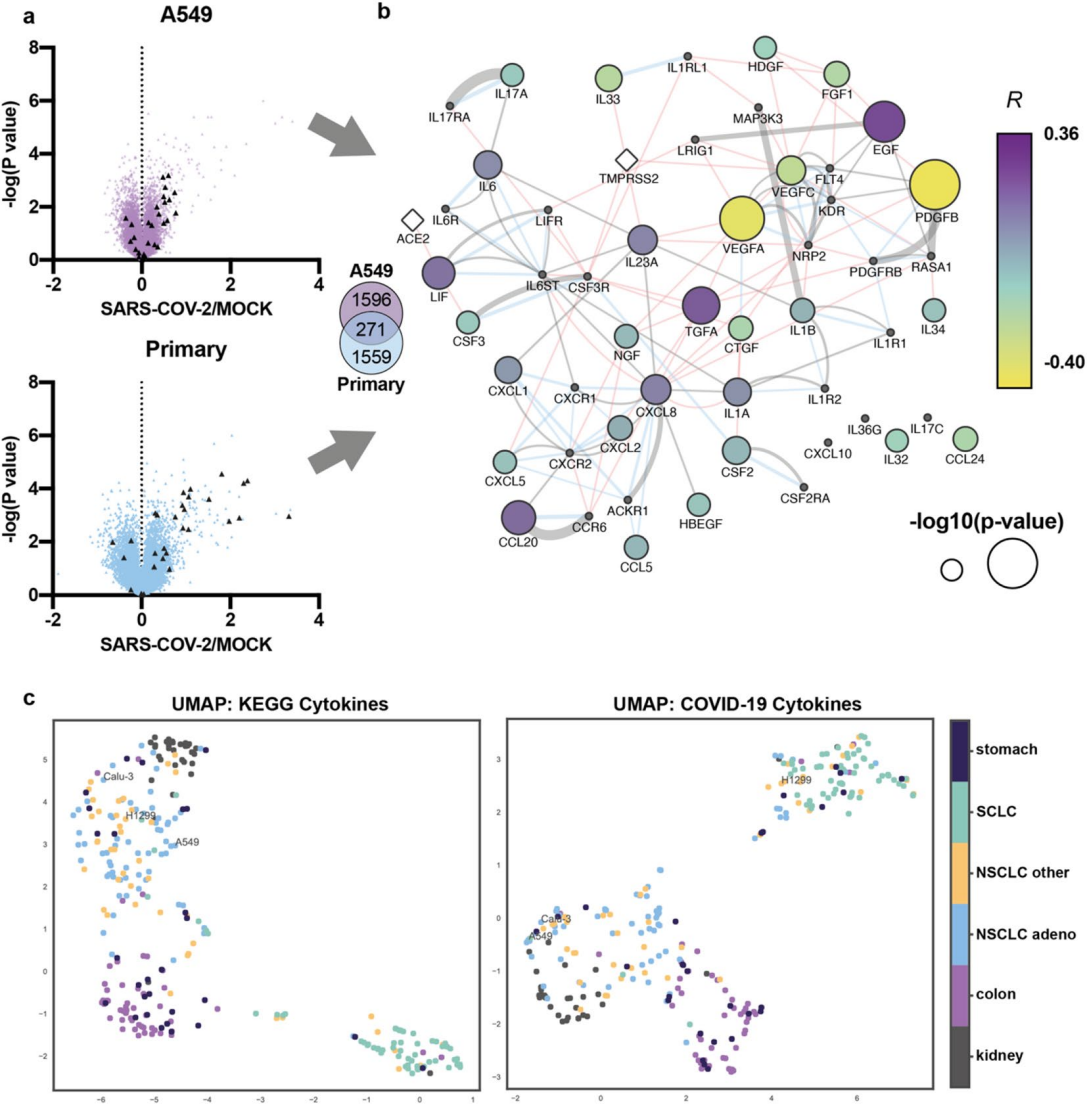
SARS-CoV-2 infection causes changes in the expression of cytokine and growth factors-coding genes that are differently connected with ACE2. (**a**) Volcano plots showing the difference between the expression of genes in A549 and primary lung cells infected with SARS-CoV-2 compared to mock-treated control and the − log(P-value). Each triangle represents a gene. Cyto-DEGs are colored in dark grey. Venn diagram illustrates number of unique A549 and NHBE (primary) DEGs and their intersection (271) (**b**) The network of proteins comprised of cytokine- and growth factors (nodes) created via GeneMania. Black nodes—genes that are added by GeneMania or are absent from GEO: GSE47460 (*CXCL10*, *IL36G*, *IL17c)*. Color indicates Spearmen correlation coefficient *R* between each gene and *ACE2* in 108 normal lung samples (purple—max correlation value, *R* = 0.36*,* yellow*—*max anticorrelation value, *R* = 0.40*)*, size—− log10 p-value of the calculated correlations. Connections show pathways (grey), genetic interaction (red), and physical interaction (blue). (**c**) UMAP analysis of 65 non-small cell lung cancer (NSCLC) adenocarcinoma, 44 NSCLC of other origins, 59 small cell lung cancer (SCLC), 31 kidney cancer, 50 colon cancer, and 28 stomach cancer cell lines from Sanger dataset. UMAP was performed using cytokine-cytokine receptor interaction pathway (KEGG) and COVID-19 cytokines gene sets.

The implication of host growth factor signaling in SARS-CoV-2 infection of cells was recently described for the first time^14^. Previously, it was shown that SARS-CoV-2 infection causes cytokine-coding genes overexpression in vitro^15^. To reveal particular cytokines and growth factors implicated in the lung cells response to SARS-CoV-2 infection we utilized recently published RNA-sequencing data of immortalized cancer human alveolar type II (A549) and primary human bronchial epithelial (NHBE) cells infected with the virus (GEO ID: gse147507). We observed 1867 differentially expressed genes (DEGs) in A549 cells infected with the SARS-CoV-2 virus compared to mock-treated cells and 1830—in NHBE cells (Fig. 1a, Supplementary Table S1). Notably, among 271 mutual DEGs we found genes coding proinflammatory proteins CXCL8 and CSF2, their expression elevated in response to SARS-CoV-2 infection.

We formed a network composed of genes coding cytokines and growth factors found among SARS-CoV-2 DEGs (cyto-DEGs) (Fig. 1b). In total, 31 cyto-DEGs were found either in A549 or NHBE cells in response to SARS-CoV-2 infection. Using the Genemania algorithm in Cytoscape we created a network-based in protein interactions (physical, genetic, or pathway) of cyto-DEGs with the addition of SARS-CoV-2 cellular receptors—ACE2 and TMPRSS2. To reveal any existing interplay between cytokines and growth factors and ACE2 in lung cells, we calculated the correlation between each input gene in the network and *ACE2* expression in the 108 samples (GEO: GSE47460) from lungs of healthy donors (Supplementary Table S1)^16^. The highest statistically significant correlation (*R* = 0.36) was between mRNA levels of epidermal growth factor *EGF* and *ACE2*. Also, *ACE2* correlated with genes coding EGFR ligand—*TGFa*, chemokines—*CCL20*, *CXCL8,* and *LIF* on mRNA level. *PDGFB* and *VEGFA* had the highest value of negative correlation with *ACE2.* In the same way, we calculated the correlation between proteins-network nodes and *TMPRRS2* expression (Supplementary Table S1). Supposedly, ACE2 is directly connected with the existence of inflammatory-primed phenotype in lung cells connected with elevated *EGF*, *FGF1*, *TGFa*, *CXCL8*, *CCL2,* and *LIF* expression.

The response of stable cell lines to SARS-CoV-2 infection in vitro highly depends on the selection criteria, which makes the study of COVID-19 complications such as CRS and drug discovery in vitro is challenging. To solve this issue, we decided to compose a panel of cytokine coding genes that could be involved in COVID-19 severity (COVID-19 cytokines). In this panel we included SARS-CoV-2 cyto-DEGs, the genes coding cytokines that are elevated in humans with severe COVID-19 obtained by manual data collection from published studies and SARS-CoV-2 receptors *ACE2* and *TMPRSS2*—in total 55 genes (Supplementary Table S2)^3,17–24^.

We aimed at understanding how the widely used in COVID-19 research cell lines derived from lung, stomach, kidney, and colon tissues differ based on the basal transcriptomic activity of particular genes. We performed UMAP^25^ analysis of 277 cancer cell lines of different origin^26^ and called it CellMAP using 2 gene sets: cytokine–cytokine receptor interaction pathway (KEGG) and COVID-19 cytokines (Supplementary Table S2). Here we provide evidence that lung cancer cell lines A549 and Calu-3 widely used in COVID-19 research have similar cytokine signatures and COVID-19 transcriptomic landscapes while H1299 cell line is distinct from them (Fig. 1c).

### Transcriptomic signature dictates the type of response to the drug

The development of fibrosis is not a unique feature of SARS-CoV-2, SARS-CoV, and MERS-CoV infection. In some patients suffering from IPF, the presence of viruses EBV, CMV, HHV-7, and HCV determines the severity of the disease^27,28^. However, it is still not clear whether viruses directly trigger the formation of fibrosis themselves or act as the co-factors in the preconditions required for the development of the disease by the secondary factors. Molecular predispositions, for example, based on the basal level of cytokine expression, could increase susceptibility of individuals to fibrosis and respiratory failure irrespective of the initiating factors^29,30^.

**Figure 2:**
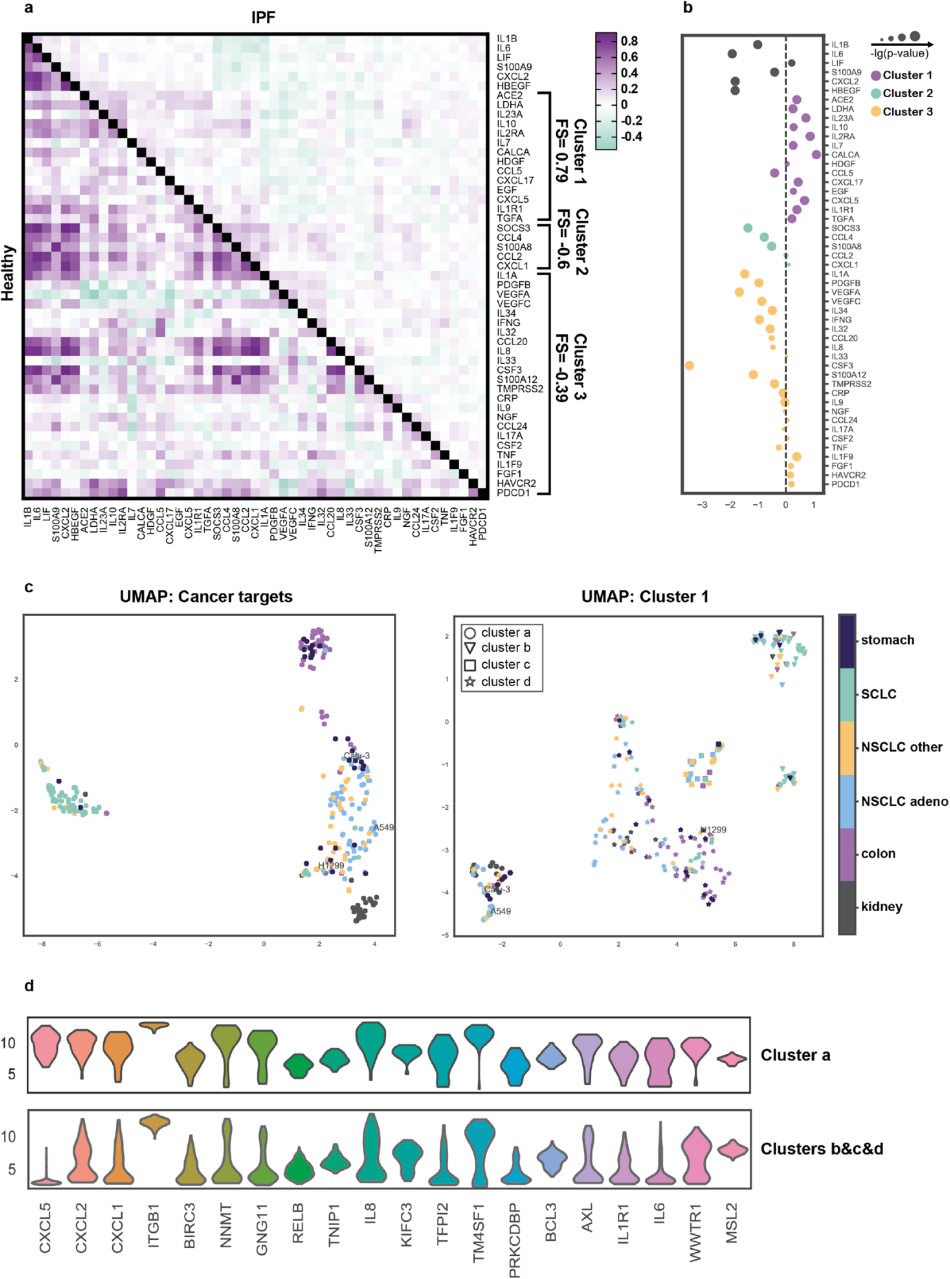
Fibrosis scoring of SARS-CoV-2 associated cytokines. (**a**) Correlation matrixes of 49 SARS-CoV-2 associated cytokine coding genes expression in the healthy lung (lower-left half) and IPF (upper-right half) samples. Pearson correlation coefficient was calculated for each gene pair and genes were clustered by Ward’s method and then bootstrapped. Fibrosis scores (FS) are indicated for each cluster. (**b**) The difference in expression of genes in healthy lung samples and IPF. (**c**) UMAP analysis of 65 non-small cell lung cancer (NSCLC) adenocarcinoma, 44 NSCLC of other origins, 59 small cell lung cancer (SCLC), 31 kidney cancer, 50 colon cancer, and 28 stomach cancer cell lines from Sanger dataset. UMAP was performed using cancer-related drug targets (Cancer targets) gene set and 14 genes from Cluster 1. Cells were then clustered (clusters a–d) using HDBSCAN algorithms due to their proximity after UMAP analysis by Cluster 1 genes. (**d**) Differentially expressed genes for *Cluster a* and *Clusters b* & *c* & *d*.

We calculated the pairwise correlations for gene expression of COVID-19 cytokines (Supplementary Table S2) in the panel of 160 IPF samples from GSE47460 dataset^16^ (49 genes were present in the dataset). To investigate the implementation of particular cytokines or their groups we performed the clusterization based on the correlation score and in the bootstrap analysis, we revealed 3 main clusters with 5–24 members (Fig. 2a and Supplementary Fig. S1a). We applied the same analysis saving the order of genes for normal lung tissue (108 samples) and lung cancer (489) (GSE3141, GSE19804, GSE2109, GSE18842, GSE33532, GSE19188, GSE43580). We observed a high number of correlating genes in normal lung samples, which lowered in IPF and lung cancer patient samples (Fig. 2a and Supplementary Fig. S1b, Supplementary Table S3).

Next, to estimate the involvement of gene groups in the fibrosis we calculated the fibrotic score (FS) of each cluster. Briefly, we calculated the difference between the number of genes that have significantly higher and lower expression in IPF samples compared to normal lung and divided this value by a total number of genes in a cluster. Among others, cluster 1 is represented by 14 genes—*ACE2, LDHA, IL23A, IL2RA, IL10, IL7, CALCA, HDGF, CCL5, CXCL17, EGF, CXCL5, IL1R1*, and *TGFA* possessed the highest FS = 0.79 (Fig. 2a). Notably, both ligands of EGFR were within this cluster 1 supporting the evidence of implementation of EGFR signaling in fibrosis development. All genes presented in cluster 1 except for CCL5 and HDGF were elevated in IPF patients compared to healthy individuals (Fig. 2b). Interestingly, *ACE2* was placed in *Cluster 1*, while *TMPRSS2 in Cluster 3*.

We decided to perform additional UMAP analysis^25^ of 277 cancer cell lines^26^ using cancer-related drug targets (COSMIC Cancer Gene Sensus and Drug–Gene Interaction database, Supplementary Table S2) and *Cluster 1* cytokines. Additionally, we asked whether the genes included in *Cluster 1* better differentiate cancer cell lines based on the transcriptomic landscape. Here we provide evidence that A549 and H1299 are likely to have similar cancer-related signatures, but different COVID-19 related profibrotic transcriptomic landscape (Fig. 2c). Calu-3 and A549 cells were found in *Cluster a*, which is defined by the differential expression of genes coding profibrotic and proinflammatory cytokines (Fig. 2d, Supplementary Table S4). Further, we ran UMAP algorithm on 7 datasets covering 489 cancer and 363 healthy lung samples. With the input of *Cluster 1* cytokines, we revealed a distinct cluster of lung cancer samples with the reduced expression of *EGFR* and *CD274* (Supplementary Fig. S1c, Supplementary Table S4). Thus, our findings suggest that transcriptomic predisposition could dictate the proviral or proinflammatory response of the cell lines and potentially lung cancer patients. Performing such analysis could be beneficial in selecting suitable models for the particular analysis based on the experiment design and for the prediction of lung cancer patients response to SARS-CoV-2 infection.

### Viral fibrotic scoring predict antiviral and antifibrotic action of the EGFR/ErbB inhibitors

**Figure 3:**
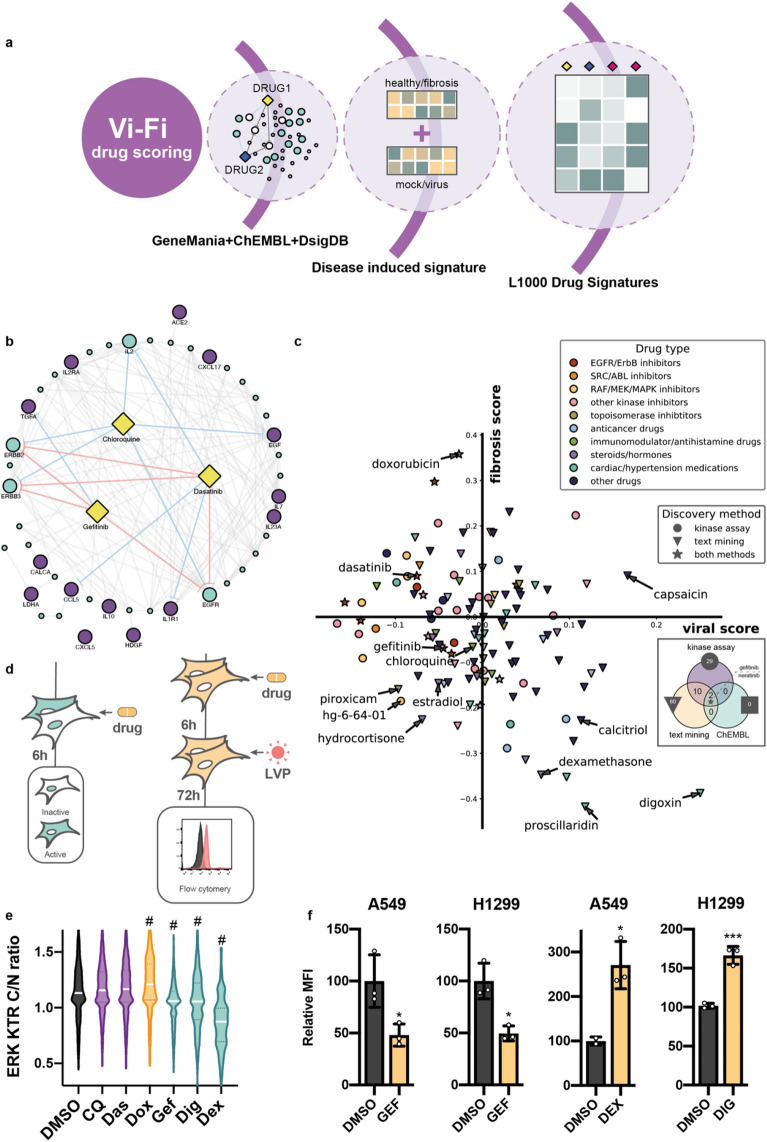
Vi-Fi drug repurposing and Vi-Fi evaluation in vitro. (**a**) Scheme of the method. (**b**) GeneMania network of potential drug targets relative to our panel of 14 SARS-CoV-2 profibrotic genes (violet nodes) and 32 additional genes predicted by GeneMania (blue nodes). A full list of genes is provided in Supplementary Table S4. The predicted interaction of gefitinib (GEF), dasatinib (DAS), and chloroquine (CQ) with proteins in the network. Red lines indicate direct inhibition determined by kinase activity assays and the blue lines—predicted inhibitory (text mining). (**c**) Scatter plot with predicted effects of drugs on SARS-CoV-2 infection (viral score) and fibrosis development (fibrotic score). Types of drugs are marked by colors concerning their medical usage, the primary target, or general mechanisms of action. Circles—drugs with proven inhibitory activity against at least one protein in the network (ChEMBL or DSigDB), diamonds—text mining (DSigDB), and stars—both methods. Venn diagram represents number of drugs available at L1000 revealed by each method. (**d**) Schemes of the experiments. (**e**) Violin plots of ERK activity in A549 ERK-KTR cells. Cells were treated with 5 µM CQ, 25 nM DAS, 25 nM doxorubicin (DOX), 5 µM GEF, 25 nM digoxin (DIG) and 10 µM dexamethasone (DEX) for 6 h. (**g**) Relative MFI of cells treated with 5 µM GEF, 10 µM DEX, and 25 nM digoxin (DIG). P-value is calculated relative to DMSO-treated sample. (**f**) Relative MFI of cells treated with GEF, DEX, and DIG. Error bars represent standard deviation from 3 independent biological replicates. *LVP* lentiviral particles. p-value for e was calculated by Mann–Whitney test, ^#^p-value < 0.001. p-value for (**f**) was determined by unpaired t-test, *p-value < 0.1, ***p-value < 0.001.

We believe that predicting viral- and fibrotic-related effects of the drug could be beneficial in drug-repurposing. Thus, we developed Vi-Fi (Viral Fibrotic) scoring algorithm of the drugs based on their L1000 signatures (http://amp.pharm.mssm.edu/l1000fwd/#) and their potential to reverse viral- and fibrosis-induced changes in gene expression (Fig. 3a)^31^. First, we addressed the issue of whether the cluster with the highest FS could be targeted by any drug with a known mechanism of action. Taking into account that drug action could be indirect, we utilized the GeneMania approach to expand our panel by the addition of proteins known to physically and genetically interact with cluster elements (Fig. 3b, Supplementary Table S4).

Then we selected drugs that can target any of the 45 proteins identified by GeneMania, by screening drugs and their targets from ChEMBL (https://www.ebi.ac.uk/chembl/) and DSigDB (http://dsigdb.tanlab.org/DSigDBv1.0/) databases (Supplementary Table S5). We found 109 drugs with described mechanisms in the ChEMBL database, 149 approved drugs, and tested kinase inhibitors from DSigDB, and 445 drugs with possible inhibitory effects based on text mining data from DSigDB collection of computational drug signatures (Supplementary Table S5). 4 compounds that were identified in the analysis of all three databases were EGFR/ErbB inhibitors. To differentiate which of these drugs could exhibit antiviral and antifibrotic action we extracted drug-induced gene expression signatures for A549 cells from the L1000 dataset and performed Vi-Fi scoring. Briefly, to calculate scores for each drug we compared its signature with genes upregulated and downregulated genes during IPF (for fibrotic score) or SARS-CoV-2 infection (for viral score). Negative score values represent antiviral or antifibrotic action, while positive scores represent proviral or profibrotic effects. A detailed description is provided in the Methods section.

In total, 121 identified drugs were present in the L1000 dataset, and 99 of them had either antiviral or antifibrotic scores (Fig. 3c, Supplementary Table S6). Most EGFR, SRC (including dasatinib), RAF (such as BRAF inhibitor HG-6-64-01), and MEK inhibitors displayed antiviral effects, while the majority of hormones, steroid hormones, immunomodulators and various drugs used to treat hypertension or heart conditions showed distinct antifibrotic scores. 34 drugs had both predicted antiviral and antifibrotic effect and 25 of these drugs had either EGFR or ERBB2/3 as one of their reported targets (Supplementary Table S6). Among FDA-approved drugs EGFR inhibitor gefitinib, nonsteroidal anti-inflammatory drug piroxicam, alkylating anticancer drug thiotepa, female hormone estradiol and steroid hormone hydrocortisone had the most prominent antiviral and antifibrotic scores. Interestingly, piroxicam, estradiol, and thiotepa had reported activity against EGFR or ERBB2. Several cardiac glycosides such as digoxin and proscillaridin while have been predicted by us and reported antifibrotic activity^32^ displayed high proviral scores. We also found that the widely used anticancer drug doxorubicin had the highest predicted profibrotic score, indicating the need for its safety evaluation for use in the treatment of cancer patients with COVID-19. Only, two drugs were revealed by the analysis of all three datasets (kinase assay, text mining and ChEMBL)—gefitinib and neratinib, both of them target EGFR. Our analysis revealed chloroquine, but its antiviral and antifibrotic scores were relatively low, which is consistent with recent reports^33^. Notably, chloroquine was identified exclusively by text mining. To expand our analysis, we used Vi-Fi scoring to predict the combined effect of each drug pair (7260 combinations) from 121 drugs identified earlier (Supplementary Table S6). For this, we combined drug-induced gene expression signatures and calculated Vi-Fi scores. We revealed several drugs, which when are used in combination with other drugs results in the majority of combinations having both antiviral and antifibrotic scores (Supplementary Fig. S2a and Supplementary Table S6). Top 10 such drugs include HSP90 inhibitor geldanamycin, IGF-1 and insulin receptor inhibitor BSM-536924, antibiotic thiostrepton, ALK inhibitor NVP-TAE684, two RAF inhibitors (hg-6-64-01 and AZ-628), two PI3K inhibitors (wortmannin and GDC-0941), anticancer drugs gemcitabine and gefitinib (Supplementary Fig. S2a). Next, we compared the results of our Vi-Fi scoring algorithm with commonly used for drug discovery and repurposing CMap algorithm^34^ (Supplementary Table S6). CMap also predicted antiviral activity of SRC inhibitors bosutinib and saracatinib, but in general, CMap showed less consistent results than Vi-Fi scoring for the antiviral effect of EGFR and ERBB2/3 inhibitors, as well as for antifibrotic action of anti-inflammatory and anti-histamine drugs (Supplementary Fig. S2b). CMap analysis predicted the absence of proviral activity of digoxin, although it predicted its antifibrotic activity.

To get an experimental evaluation of the Vi-Fi algorithm we used previously developed in our laboratory system based on the transfer of gene coding fluorescent protein into a host cell by the lentiviral vector particles^13^. We selected two lung cancer cell lines with similar cancer phenotypes and different COVID-19 related transcriptomic signatures based on our CellMAP analysis. Considering the vital role of the MAPK/ERK pathway in controlling processes in host cells including cytokine signaling^35^ we decided to study its implication in the response of lung cells to drugs with different viral and fibrotic activity. We utilized a kinase translocation reporter (ERK-KTR) to track the ERK activity in H1299 and A549 cells at the single-cell level. Briefly, EKR-KTR allows quantification of ERK activity in a single cell based on fluorescent reporter translocation between nucleus and cytoplasm^36^. For this, H1299-KTR and A549-KTR cells were with non-toxic concentrations of 5 drugs with different fibrotic potential—EGFR inhibitor gefitinib, heart glycoside digoxin, corticosteroid dexamethasone, anticancer antibiotic doxorubicin, and SRC inhibitor dasatinib as shown in Fig. 3d. Gefitinib, digoxin, and dexamethasone inhibited ERK activity while doxorubicin activated it in A549 cells (Fig. 3e). Dexamethasone also inhibited ERK in H1299 cells (Supplementary Fig. S3a,d). Thus, our findings show that the drugs with ERK inhibitory activity possess an antifibrotic potential and ERK monitoring could be beneficial to predict the potential to prevent fibrosis. As the utilization of the Vi-Fi scoring predicted the antiviral potential of EGFR/ERbB inhibitors including gefitinib, chloroquine, and dasatinib, along with a high proviral capacity of dexamethasone and digoxin, we pretreated A549 and H1299 cells with these agents before the addition of LVP as shown on Fig. 3e. Gefitinib, chloroquine, and dasatinib significantly reduced the efficacy of lentiviral transduction of A549 and H1299 cells while digoxin and dexamethasone elevated the transduction rate of the cells (Fig. 3f, Supplementary Fig. S3b,c).

### Antifibrotic drugs change the response of lung cells to EGF

As Vi-Fi scoring predicted strong dual antifibrotic and antiviral potential of EGFR inhibitors and we showed the ability of gefitinib to downregulate ERK activity along with the reduction of lentiviral transduction efficacy in lung cells pretreated with this drug we decided to understand how EGFR downstream signaling is implicated in this process.

**Figure 4:**
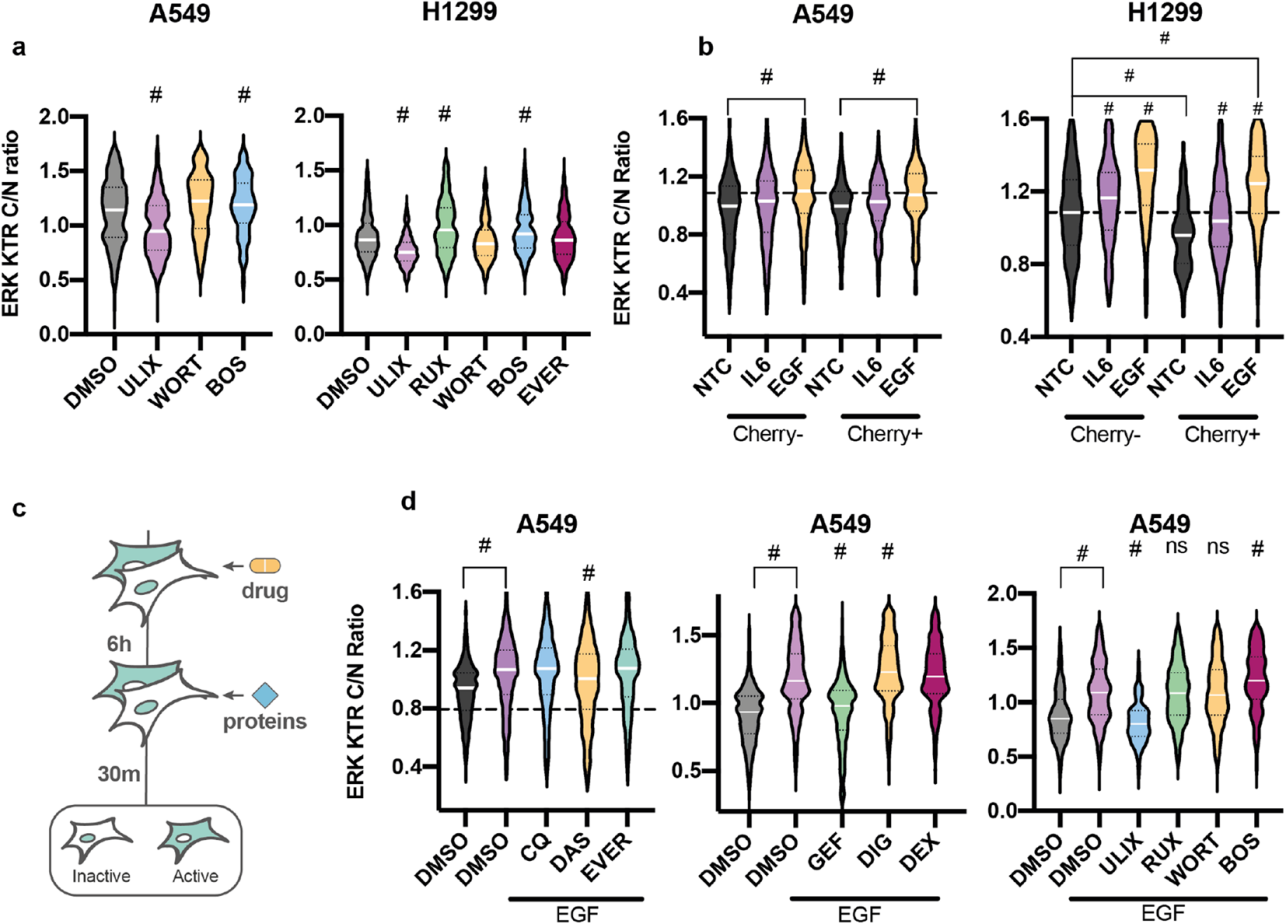
Drugs and proteins change the transduction efficacy of lung cells and the ERK activity. (**a**) Violin plots of ERK activity in A549 and H1299 cells measured with ERK-KTR. A549 cells were treated with 50 nM ulixertinib (ULIX), 10 µM wortmannin (WORT), 1 µM bosutinib (BOS) for 6 h. H1299 cells were treated with 50 nM ulixertinib (ULIX), 10 µM ruxolitinib (RUX), 10 µM wortmannin (WORT), 1 µM bosutinib (BOS), and 100 nM everolimus (EVER) for 6 h. p-value is calculated relative to DMSO-treated sample. (**b**) Violin plots of ERK activity in A549 and H1299 cells with ERK-KTR. Cells were incubated with lentiviral particles for 72 h and proteins were added to serum negative media 30 min before the fluorescence detection. (**c**) Scheme of the experiment: A549 and H1299 cells with ERK-KTR were treated with the drugs and proteins for 6 h in serum negative media before the addition of 100 ng/mL EGF for 30 min before fluorescence detection. (**d**) Violin plots of normalized ERK activity in A549 cells measured with ERK-KTR. Cells treated with drugs were compared to cells treated only with EGF by Mann–Whitney test. Drugs and proteins concentrations: 5 µM chloroquine, 25 nM dasatinib, 100 nM everolimus, 5 µM gefitinib (GEF), 25 nM digoxin (DIG), 10 µM dexamethasone (DEX), 50 nM ulixertinib (ULIX), 2.5 µM ruxolitinib (RUX), 10 µM wortmannin (WORT), 1 µM bosutinib (BOS), 100 ng/mL IL6 and EGF. p-value is calculated relative to DMSO + EGF treated sample unless it is marked otherwise. *LVP* lentiviral particles. p-value for (**a**–**d**) was calculated by Mann–Whitney test, ^#^p-value < 0.001.

By utilization of ERK-KTR reporter and the lentiviral vector-based system, we measured the antiviral and antifibrotic potential of the drugs known to inhibit EGFR downstream kinases: mTORC1, JAK2, SRC and MAPK1/2—everolimus, ruxolitinib, bosutinib and ulixertinib (Fig. 4d and Supplementary Fig. S4a). Ulixertinib blocked ERK activity and ruxolitinib activated it in both A549 and H1299 cells (Fig. 4a). Bosutinib stimulated ERK activity in H1299 cells but not in A549 (Fig. 4a).

We also measured the impact of IL6 and EGF stimulation on transduced and non-transduced cells. EGF activated ERK in both A549 and H1299 cell lines and the action was more prominent on the transduced population of H1299 cells (Fig. 4b). IL6 stimulated ERK activity only in H1299 cells. We also detected that ERK activity was decreased in transduced H1299 and A549 cells compared to non-treated cells, and there was a bystander decrease of ERK activity in non-transduced A549 cells. Next, we decided to understand whether any of the tested drugs reverse EGF-mediated ERK activity. For that we pretreated A549 and dasatinib, gefitinib and ulixertinib blocked EGF-driven ERK activation in A549 cells (Fig. 4c,d and Supplementary Fig. S4b).

Next, we measured the ability of cellular kinase targeting drugs (everolimus, ruxolitinib, ulixertinib) to affect the transduction rate of A549 and H1299 cells. Everolimus significantly reduced the transduction rate of both cells, ruxolitinib stimulated it only in H1299 cell line and ulixertinib had no impact on the transduction efficacy (Supplementary Fig. S4c). However, no significant changes in the transduction rate of A549 or H1299 cells were detected with the addition of EGF (Supplementary Fig. S4d). Next, we treated the cells with EGF along with the addition of drugs with promising antiviral action: chloroquine, dasatinib, and everolimus to test the possibility of EGF to modulate the drug response. Our data demonstrate that the addition of EGF results in a dramatic increase of mean fluorescent intensity of the A549 cells treated with dasatinib or everolimus and H1299 treated with chloroquine (Supplementary Fig. S4d). Our results thus confirm that EGF indeed is involved in the infection processes of the lung cells and EGFR signaling is a potential drug target in COVID-19 treatment.

### EGF induces *ACE2* expression and proinflammatory change in lung cells

**Figure 5:**
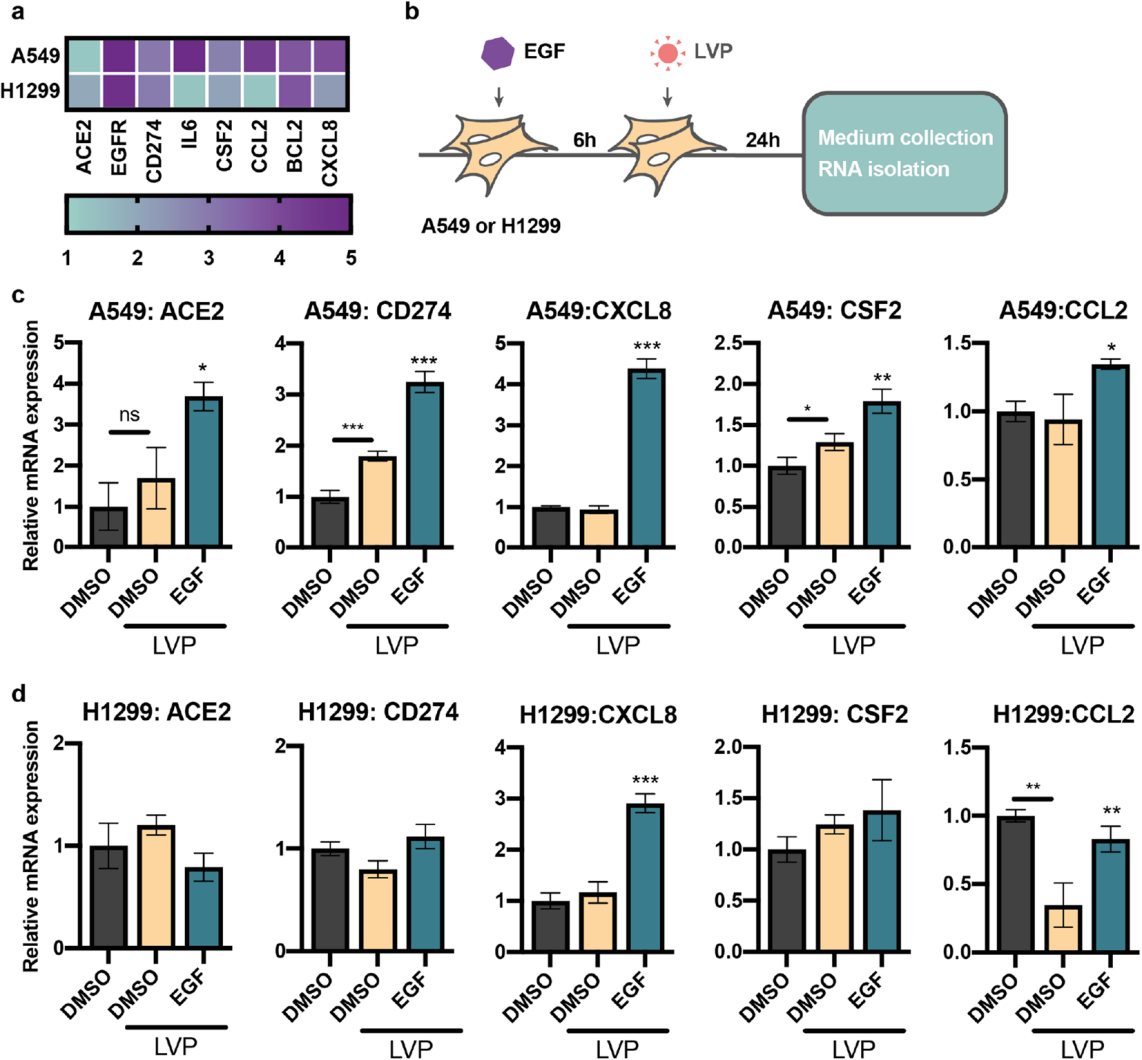
EGF activates proinflammatory signature in A549 cells. (**a**) Heatmap representing relative to GAPDH mRNA expression level of genes in nontreated A549 and H1299 cells. (**b**) Scheme of the experiment: A549 and H1299 cells were treated with EGF (100 ng/mL) for 6 h followed by the addition of lentiviral particles (LVP). The medium was collected and RNA was isolated 24 h later. Relative mRNA level of ACE2, CD274, CXCL8, CSF2, and CCL2 genes in A549 (**c**) and H1299 (**d**) cells treated with EGF (100 ng/mL).

First, we measured the mRNA levels of *ACE2* and *TMPRSS2*, *EGFR*—EGF receptor, *IL6*, *CXCL8* and *CCL2*—proinflammatory cytokines and chemokines, *BCL2*—apoptosis regulator, and *CD274*—programmed cell death ligand in A549 and H1299 cells. We decided to measure *CD274* as the exhaustion of CD8 + T cells with the elevation of PD-1 (programmed cell death protein 1, also known as CD279) expression is present in severe COVID-19 patients^37,38^. In these cells we detected comparable levels of *ACE2*, *EGFR*, *CD274*, *BCL2*, *CSF1,* and *CSF2* mRNA expression, but the levels of *IL6*, *CXCL8,* and *CCL2* were significantly higher in A549 cells, suggesting the existence of the inflammation-primed transcriptomic activity in this cell line (Fig. 5a). These results are consistent with the results of UMAP analysis which showed distinct COVID-19 related transcriptomic signatures of A549 and H1299 cells (Fig. 2c). Also, we detected a very low expression level of *TMPRSS2* mRNA in both cell lines.

As a next step towards understanding the mechanisms of EGFR-signaling implementation in the COVID-19-related severity, we stimulated A549 and H1299 cells with the recombinant EGF before the addition of lentiviral particles (Fig. 5b). The lentiviral transduction of A549 cells led to the elevation of the *CD274* and *CSF2* mRNA expression, also *ACE2* expression had an upcoming trend (Fig. 5c,d). We also found that *CD274* expression was elevated in A549 cells treated with SARS-CoV-2 virus compared to mock-treated cells (Supplementary Table S1) and in virus infected lung cells of COVID-19 patients^39^. We found strong elevation of the expression of the genes coding ACE2, CD274, CXCL8, CCL2 and CSF2 in the A549 cells that was not detected in H1299 cells. Notably, only *CXCL8* up-regulation was common for both cell lines.

Bronchoalveolar fluid (BALF) from patients with severe COVID-19 was shown to be enriched in chemokines that are responsible for the attracting of inflammatory monocytes^40^. Therefore, we investigated the response of human model macrophages derived from THP-1 cell line to the conditioned culture medium harvested from pretreated A549 and H1299 cell lines (Supplementary Fig. S5a). The conditioned culture medium of the cells transduced with lentiviral particles induce the expression of *CXCL8* in macrophages (Supplementary Fig. S5b,c)*.* Chemokine CXCL8 is crucial for the attraction of macrophages and neutrophils into the inflamed organs, including lung. At the same time *CSF2* and *IL6* mRNA expression had an uprising trend in macrophages treated with H1299 conditioned medium.

## Discussion

Within the present study, we have focused on the host cell inflammatory response to the SARS-CoV-2 infection. We suggested that not only immune cells, but the host lung cells themselves, are involved in the development of fibrosis and CRS. We aimed at the characterization of molecular mechanisms defining severity of COVID-19 to reveal novel drug targets involved in fibrosis and viral infection. In general, the current work shed light on the implementation of growth factor signaling in the COVID-19 related fibrosis. With a combination of the computational and molecular biological approaches we developed a novel strategy for drug repurposing and validated it in vitro.

We developed the drug repurposing approach that overcomes the limitations of the recently proposed methods to predict effective anti-SARS-CoV-2 drugs^12^. These computational methods are either based on drug interaction with virus-associated protein networks or on drug-induced transcriptomic signatures, and mostly focus only on antiviral properties of the compound. We developed *Vi-Fi scoring* based on the protein network associated with both SARS-CoV-2 infection and pulmonary fibrosis followed by the quantifying drug antiviral and antifibrosis scores based on their transcriptomic signatures. This allowed us to narrow down drugs with high probability of dual antiviral and antifibrotic effect. The ability to predict drug combinations makes Vi-Fi scoring even more powerful. Development of the combinational therapeutic approaches are of the particular interest for SARS-CoV-2 treatment as well as in the field of anticancer drug discovery, as the monotherapy often is a source of drug resistance. Even the development of high-throughput platforms for drug screening have not significantly simplified the combinational drug discovery because of the innumerable possible drug combinations. We demonstrate that Vi-Fi scoring could be easily modified to predict drug combinations. Thus, our Vi-Fi scoring algorithm is a flexible tool and possesses a great potential to reveal drugs and their combinations that target multiple pathological processes in any disease. With the Vi-Fi approach we revealed that anticancer drugs such as EGFR/ErbB inhibitors possess antiviral and antifibrotic potential. This finding was promising as the issue of chemotherapy adaptation during the COVID-19 pandemic is unsolved. Receptor tyrosine kinase inhibitors such as gefitinib effectiveness in our experiments is also consort with the lung cancer data as patients with EGFR inhibitors administration had lower COVID-19 severity rate^41^. Interestingly, among steroid hormones dexamethasone showed highest antifibrotic score, which explains success of it use for treatment of severe COVID-19 cases, despite dexamethasone having moderate proviral score and proviral action on LVP transduction of lung cancer cells. Although our analysis has identified chloroquine as a drug with antiviral and antifibrotic drug, its connection with COVID-19 associated proteins in lung cells was revealed only by text mining compared to EGFR/ErbB inhibitors, that were present in all three databases and had higher antiviral scores than chloroquine.

For the first time, we apply single cell monitoring of ERK activity for the in vitro study of proinflammatory response of lung cells. ERK activity correlated with the profibrotic action of the tested drug suggesting that monitoring of ERK activity in lung cells can be beneficial to measure drug potential to block proinflammatory and profibrotic cytokines. Our results suggest that antifibrotic and anti-inflammatory effect of drugs is realized not only through affecting immune cells but also by inhibition of autocrine signaling in lung cells. Also, we provided an experimental and computationally expanded findings regarding the significance of transcriptomic landscape of the cell line in the development of the response. We developed an algorithm based on the machine learning to predict the similarity of cell lines. The question of model selection for in vitro studies is urgent and is not limited to COVID-19 research.

A common observation in each of our analysis is the implementation of EGF/EGFR signaling in the SARS-CoV-2 infection and proinflammatory priming of the host cells. Notably, EGFR was recently described as SARS-CoV-2 receptor, that supports our findings, even though these findings were independent and obtained by the study of different processes during COVID-19 infection^42^. Although SARS-CoV-2 associated genes such as *CCL2* and *ACE2* are known to be IFN-inducible^43^, it is likely that EGF serves a trigger of proinflammatory response in lung cells and even substitutes IFNγ. We showed that EGF induces the expression of *CXCL8*, *CSF2*, *CCL2*, *ACE2,* and *CD274* in lung cells. Notably, it was shown that recombinant IFN failed to induce a detectable level of *ACE2* expression in A549 cells and the fact has been interpreted as unsuitably of transformed lung cells to be a model for SARS-CoV-2 infection studies in vitro^44^. We explain that by the absence of EGF, which plays a substantial role in the supporting SARS-CoV-2 infection and the development of the fibrosis and CRS. Also, EGF is likely implemented in the immune cells attraction to the damaged lung in the severe COVID-19 patients. Among EGF-inducible genes *CSF2* is associated with tissue damaging by the attraction of proinflammatory macrophages and also could be produced by epithelium airways cells in response to allergens^45^*.* CSF2 is known to be implicated in the attraction of proinflammatory M1 macrophages. Also, upregulation of *CD274* expression could be a mechanism underlying the protection of virus-infected cells from being eliminated by cytotoxic CD8 + T cells or driving T cells exhaustion by PD-1/PD-L1 interaction. EGFR signaling implication in SARS infection was earlier the object of potent interest, and the role of different EGFR ligands was discussed in the context of fibrosis reduction after the infection with the previously described respiratory viruses and the inhibition of growth factor signaling was recently described as a promising strategy to prevent SARS-CoV-2 infection^14,46^. Concomitant with our results it was previously reported that both EGF and IFNγ induce *CD274* expression in lung adenocarcinoma cells and act through the MAPK cascade, and imbalanced PD-1/PD-L1 interaction is observed during viral infections^47,48^.

Altogether, we provide evidence that EGF and EGF-inducible genes are involved in fibrosis and inflammation, that could be prevented by targeting EGFR/MAPK pathway by EGFR/ErbB inhibitors such as gefitinib and dasatinib and we anticipate that Vi-Fi scoring will be a valuable tool that can be rapidly adopted by those who study drug responses.

However, this study has several limitations. Development of novel therapy is a very complex multistage process; our study covers the initial steps of the identification of the potential and antiviral drugs—computational drug scoring (Vi-Fi) and in vitro validation. We believe that our data will encourage further experimental validation of identified drugs including EGFR/ErbB inhibitors in animal models. Also, our scoring approach covers drugs with potential antifibrotic and antiviral activity, what makes it a powerful tool for drug repurposing that can be applied to other diseases. Another limitation is that SARS-CoV-2-induced inflammatory long-term response that triggers multiple organs cannot be fully modeled in short-term in vitro experiments. By the utilization of ERK activity reporter and cell lines of two origins (lung and hematopoietic) we were able to register the response similar to initiating events of the ongoing cytokine storm and viral infection that arises in host cells.

## Material and methods

### Cell culture and drugs

Human lung cancer cells H1299^49^ and A549 (DSMZ ACC 107; Deutsche Sammlung von Mikroorganismen und Zellkulturen GmbH, Braunschweig, Germany) and embryonic kidney cells HEK-293T were cultured at 37 °C and 5% CO_2_ in DMEM growth medium supplemented with 10% fetal bovine serum (FBS). THP-1 (ATCC TIB-202) cells were cultures in RPMI-1640 growth medium supplemented with 10% FBS. All growth media were also supplemented with 2 mM l-glutamine, 100 units/mL penicillin, 100 μg/mL streptomycin, and 1 mM sodium pyruvate. A549 and H1299 cell lines were gifted by Prof. Dr. Thomas Dobner, Heinrich-Pette Institute at the Leibniz Institute for Experimental Virology. HEK-293T cell line was a gift from Prof. Dr. Boris Fehse, UKE. THP-1 cell line was gifted Dr. Nikita Nikiforov from Institute of Experimental Cardiology, National Medical Research Center of Cardiology. RPMI-1640, DMEM, penicillin/streptomycin, sodium pyruvate, and l-glutamine were purchased from Gibco (ThermoFisher Scientific, USA). THP-1 cell were differentiated by incubation with 100 ng/mL phorbol 12-myristate 13-acetate (PMA) (P1585, Sigma Aldrich) for 24 h^50^. All cell lines were routinely checked for mycoplasma contamination by Hoechst or DAPI staining. None of the cell lines used in the study belongs to the list of misidentified cell lines. Drugs used in the current study bosutinib (PZ0192, Sigma Aldrich), chloroquine (C6628, Sigma Aldrich), dasatinib (11498, Cayman Chemical Company), everolimus (S1120, Selleckchem), gefitinib (SML1657, Sigma Aldrich), ulixertinib (S7854, Selleckhem), digoxin (2266, Cayman Chemical Company), dexamethasone (D4902, Sigma Aldrich), ruxolitinib (S1378, Selleckchem), wortmannin (S2758, Selleckchem).

### Differential expression analysis and correlation

Multiple t-tests with false discovery rate (FDR) correction for genes in SARS-CoV-2 infected A549 and NHBE cells were performed in GraphPad Prism 8 software. Transcriptomic data of A549 (6 samples) and NHBE (6 samples) cells was analyzed separately. Genes with P-value < 0.01 were considered as differentially expressed. Differential expression analysis of markers for clusters identified by UMAP was performed in Python using Mann–Whitney t-tests and FDR correction. Spearmen coefficient of correlation between genes was calculated in GraphPad Prism 8 software.

### Genemania

To visualize interactions between identified genes, we employed the GeneMANIA (http://www.genemania.org/), a Cytoscape application tool used for the functional prediction of gene and protein interactions. For network creation utilized for drug discovery physical and genetic interactions, and pathways databases were used.

### Lentiviral particles production

The stocks containing VSV-G pseudotyped lentiviral particles were generated by co-transfection of HEK293T with LeGO-iC2++ or pLentiCMV Puro DEST ERKKTRClover, and packaging plasmids. pLentiCMV Puro DEST ERKKTRClover was a gift from Markus Covert (Addgene plasmid #59150). For the creation of H1299 cells expressing ERK KTR, cells were transduced with ERKKTRClover lentiviral particles to achieve ~ 50% transduction rate and then transduced cells were selected with medium supplemented with 1 μg/mL puromycin (Sigma).

### Real-time PCR

RNA extraction from myeloid and lung cancer cells and qRT-PCR analysis was performed in accordance with manufacturer protocols as described in Ref.^51^. Primer sequences used in this study are provided in Supplementary Table S7.

### ERK KTR quantification

H1299 and A549 cells with ERK-KTR expression were generated by lentiviral transduction of pLentiCMV Puro DEST ERKKTRClover plasmid, which was a gift from Markus Covert (Addgene plasmid #59150). For nuclear segmentation cells were incubated with 500 ng/mL Hoechst-33342 for 30 min before imaging on Leica DMi8. To measure ERK activation by EGF and IL6 cells were cultured in medium without serum. Each experiment was repeated at least two times, three microscopic fields were chosen randomly for imaging for each well. Cytoplasm to nucleus ratios (C/N ratio) of mClover intensity were calculated for each cell. Nuclei and cytoplasm segmentation, and object intensity calculations were performed with CellProfiler. Median intensities of mClover fluorescence in cytoplasm and nucleus were quantified and used to calculate cytoplasm to nucleus (C/N) ratios for each cell. Outliners were removed based on z-score. Data processing was performed in Python and GraphPad Prism 8.4.

### Correlation matrixes and clustering

We used transcriptomic data for 160 IPF samples from LGR Consortium dataset GSE47460^16^ and calculated Spearmen’s rank correlation coefficients in GraphPad 8.4 for each pair of 49 COVID-19-associated cytokine genes present in the dataset. Then pairwise correlation matrix was clustered by Ward’s hierarchical cluster method and bootstrap resampling using PVclust R package^52^. Clusters with 5 or more genes and AU-value > 95 were considered as stable (Supplementary Fig. S3a). Similar correlation matrixes were calculated for 108 healthy lung samples from the same data set, 489 lung cancer and 363 control samples from 7 NCSLC datasets: GSE3141^53^, GSE19804^54^, GSE2109, GSE18842^55^, GSE33532, GSE19188^56^, GSE43580^57^.

### UMAP

Transcriptomic data for 277 lung, colon, stomach and kidney cancer cell lines was obtained from Genomics of Drug Sensitivity in Cancer dataset^58^ and UMAP analysis was performed using 4 different gene sets (Supplementary Table S2). For initial NSCLC analysis transcriptomic data from 7 previously described NCSLC datasets was used as well as 410 additional samples from GSE63074 dataset. Since GSE63074 dataset showed a significant batch effect it was removed from final analysis. UMAP was performed using Python library (https://github.com/lmcinnes/umap), appropriate codes are provided (https://github.com/CancerCellBiology/CELL-MAP). Cluster analysis for UMAP data was performed using HDBSCAN algorithms (https://github.com/scikit-learn-contrib/hdbscan).

### Cmap

Cmap analysis was performed using clue.io online platform. The top 150 differentially expressed genes identified in A549 cells in response to SARS-CoV-2 infection were used as a query. Genes used for the analysis are listed in Supplementary Table S1. We used only the drug scores calculated for A549 cell line. The Cmap scores are available in Supplementary Table S6.

### Vi-Fi scoring

Physical and genetic interactions, and pathways databases were used for network creation with addition of up to 30 related genes. ChEMBL (https://www.ebi.ac.uk/chembl/)^59^ and DSigDB (http://dsigdb.tanlab.org/DSigDBv1.0/)^60^ databases were used for initial selection of drugs that interact with at least one target from gene/protein network. From ChEMBL only drugs with described mechanisms were selected. From DSigDB FDA approved drugs and kinase inhibitors databases were used to find drugs with tested inhibitory activity. DSigDB computational drug signatures databases were used to find drugs that inhibit, decrease phosphorylation, expression and stability, block interaction with ligand and promote degradation of target genes/proteins. To calculate antiviral and antifibrotic scores for drugs we used L1000 binary drug-induced gene expression signatures in full space (http://amp.pharm.mssm.edu/L1000FWD/)^31^. Drug-induced signatures were compared with two disease specific gene expression signatures: differentially expressed genes (DEGs) in idiopathic pulmonary fibrosis (IPF) vs. healthy lung used for fibrosis score calculation, and DEGs in SARS-CoV-2 infected A549 and normal human bronchial epithelial (NHBE) cells used for viral score calculation (Supplementary Table S6). Vi-Fi scores were calculated according to this formula:

$$S = \left( {\frac{{\mathop \sum \nolimits_{i = 1}^{n} S_{i} \times BP_{i} }}{n}} \right)_{up} - \left( {\frac{{\mathop \sum \nolimits_{i = 1}^{n} S_{i} \times BP_{i} }}{n}} \right)_{down} ,$$

where S is the fibrotic or viral score for the drug; $S_{i}$ is the score for an individual gene from drug-induced signature: + 1 if gene expression is upregulated in a disease-specific signature, − 1 if downregulated; $BP_{i}$ is the Boolean p-value: 1 if p-value for gene expression difference in a disease-specific signature is < 0.05, 0 otherwise; $n$ is the number of upregulated (*up*) or downregulated (*down*) genes in drug-induced signature. Negative score values represent antiviral or antifibrotic predicted effect, while positive score represents proviral or profibrotic.

For calculation of combined drugs scores L1000 drug-induced signatures were combined and genes which expression changes in opposite directions were removed from the analysis. If the same gene was present in both drug signatures that gene was taken into account only once. All analysis were performed in Python and codes are available (https://github.com/CancerCellBiology/Vi-Fi-scoring). The interactive 2D plot is available on our web-site 6zr.ru.

### Statistics

All the data are expressed as mean ± SD from at least three individual experiments unless stated otherwise in the text. Statistical significances of differences observed in flow cytometry and ERK-KTR experiments were determined by Mann–Whitney non-parametric test. Statistical significances for real-time PCR experiments were determined by an unpaired two-sided Student t-test.

### Ethics declarations

No humans or animals were directly involved in the study.

## Supplementary Information

Supplementary Information.

Supplementary Table S1.

Supplementary Table S2.

Supplementary Table S3.

Supplementary Table S4.

Supplementary Table S5.

Supplementary Table S6.

Supplementary Table S7.

Supplementary Table S8.

## Data Availability

The results presented herein are based only on publicly available datasets. They are available using identifiers listed in Supplementary Table S8.

## Code availability

The source code used to generate main and supplementary figures is published (https://github.com/CancerCellBiology).
