# Publisher Correction: Viral fibrotic scoring and drug screen based on MAPK activity uncovers EGFR as a key regulator of COVID-19 fibrosis

**DOI:** 10.1038/s41598-021-97281-9

**Published:** 2021-08-30

**Authors:** Elmira R. Vagapova, Timofey D. Lebedev, Vladimir S. Prassolov

**Affiliations:** 1 Engelhardt Institute of Molecular Biology, Russian Academy of Sciences, Moscow, 119991 Russia; 2 Center for Precision Genome Editing and Genetic Technologies for Biomedicine, Engelhardt Institute of Molecular Biology, Russian Academy of Sciences, Moscow, Russia

Correction to: *Scientific Reports* 10.1038/s41598-021-90701-w, published online 27 May 2021

The original version of this Article omitted an affiliation for Elmira R. Vagapova. The correct affiliations for Elmira R. Vagapova are listed below:

Engelhardt Institute of Molecular Biology, Russian Academy of Sciences, Moscow, 119991, Russia

Center for Precision Genome Editing and Genetic Technologies for Biomedicine, Engelhardt Institute of Molecular Biology, Russian Academy of Sciences, Moscow, Russia

The original Article has been corrected.

