# Supplementary material for: Viral fibrotic scoring and drug screen based on MAPK activity uncovers EGFR as a key regulator of COVID-19 fibrosis
Source: Sci Rep. 2021 May 27;11:11234. doi: 10.1038/s41598-021-90701-w (PMC8160348; doi:10.1038/s41598-021-90701-w)
Supplement: Supplementary file 1 — Supplementary Information. [file 41598_2021_90701_MOESM1_ESM.pdf]

Viral Fibrotic scoring and drug screen based on MAPK activity uncovers EGFR as a key regulator of COVID-19 fibrosis

Elmira R. Vagapova, Timofey D. Lebedev, Vladimir S. Prassolov

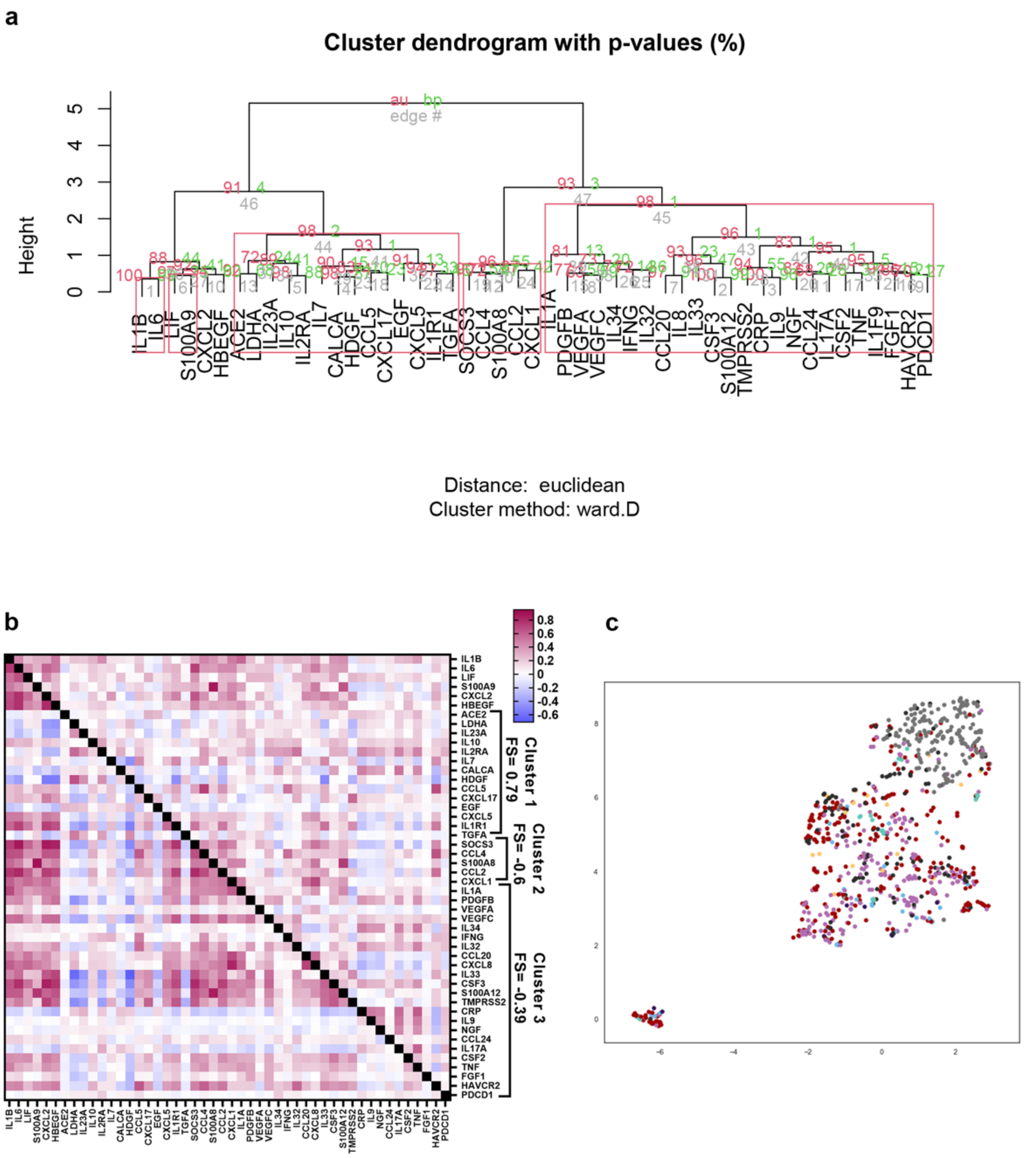

Figure S1 Clustering of COVID-19 associated genes and lung cancer samples. Related to Figure 2.

(a) Bootstrap results for clustering of COVID-19 associated genes in IPF samples. **AU** (Approximately Unbiased, red) p-value and **BP** (Bootstrap Probability, green) values are present. Bootstrap and clustering were performed using PVclust R package. Stable clusters are highlighted by red rectangles.

(b) Correlation matrixes of 47 SARS-CoV-2 associated cytokine coding genes expression in control lung (lower-left half) and lung cancer (upper-right half) samples. Person correlation coefficient was calculated for each gene pair. Fibrosis scores (FS) are indicated for each cluster.

(c) UMAP analysis of 489 lung cancer and 363 control samples from 7 lung cancer datasets described in Methods section. UMAP was performed using 14 genes from Cluster 1. Different histology types are marked by color.

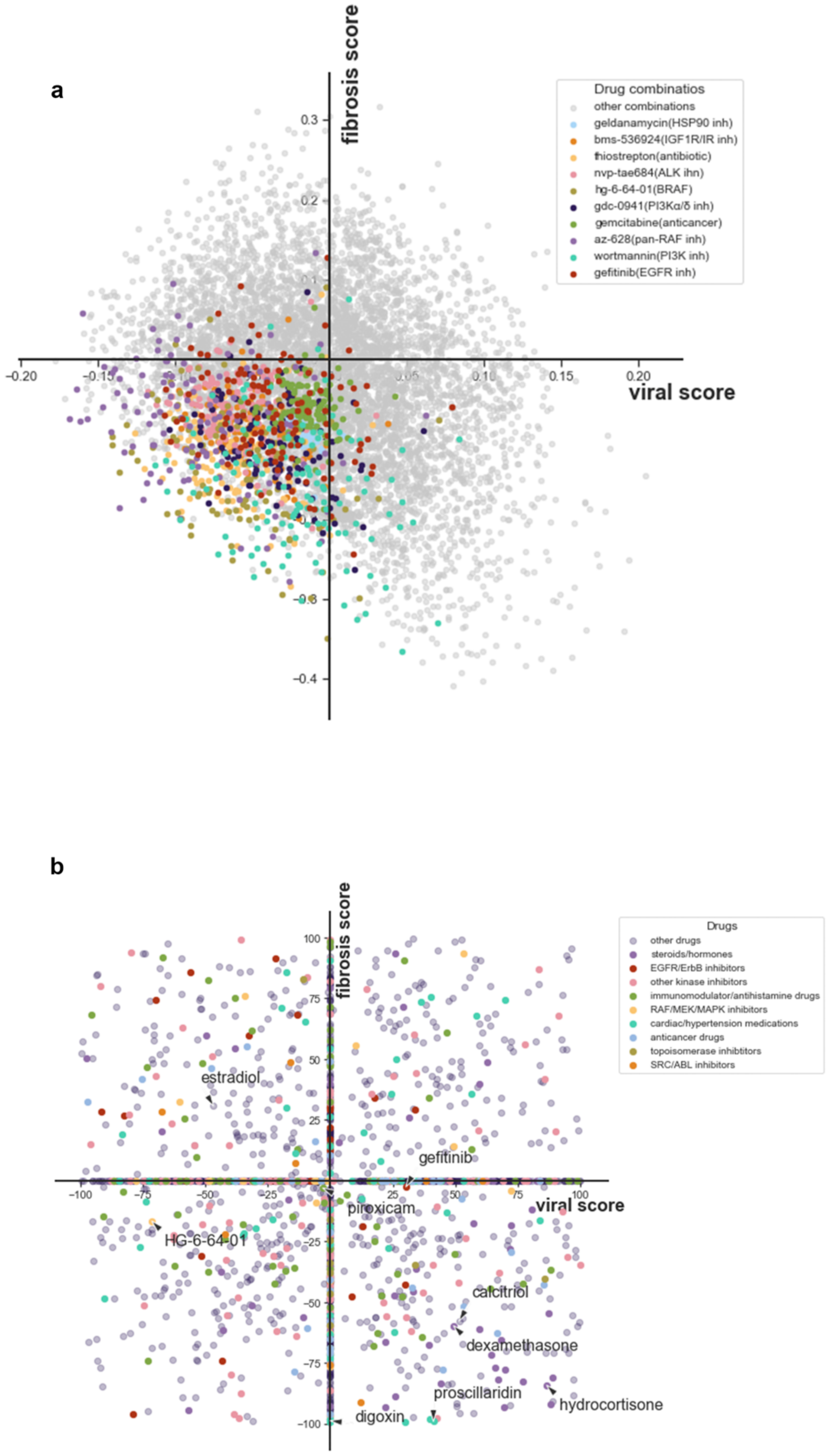

Figure S2 Drug combinations prediction scores and CMap scores. Related to Figure 3.

(a) Drug combinations scores obtained by Vi-Fi scoring algorithms. Top 10 drugs that result in antifibrotic and antiviral scores in combination with other drugs are marked by color.

(b) CMap score for 1915 drugs present in CMap database with non-zero fibrosis and viral scores. Different types of drugs are marked by colors with respect to their medical usage, primary target or general mechanisms of action.

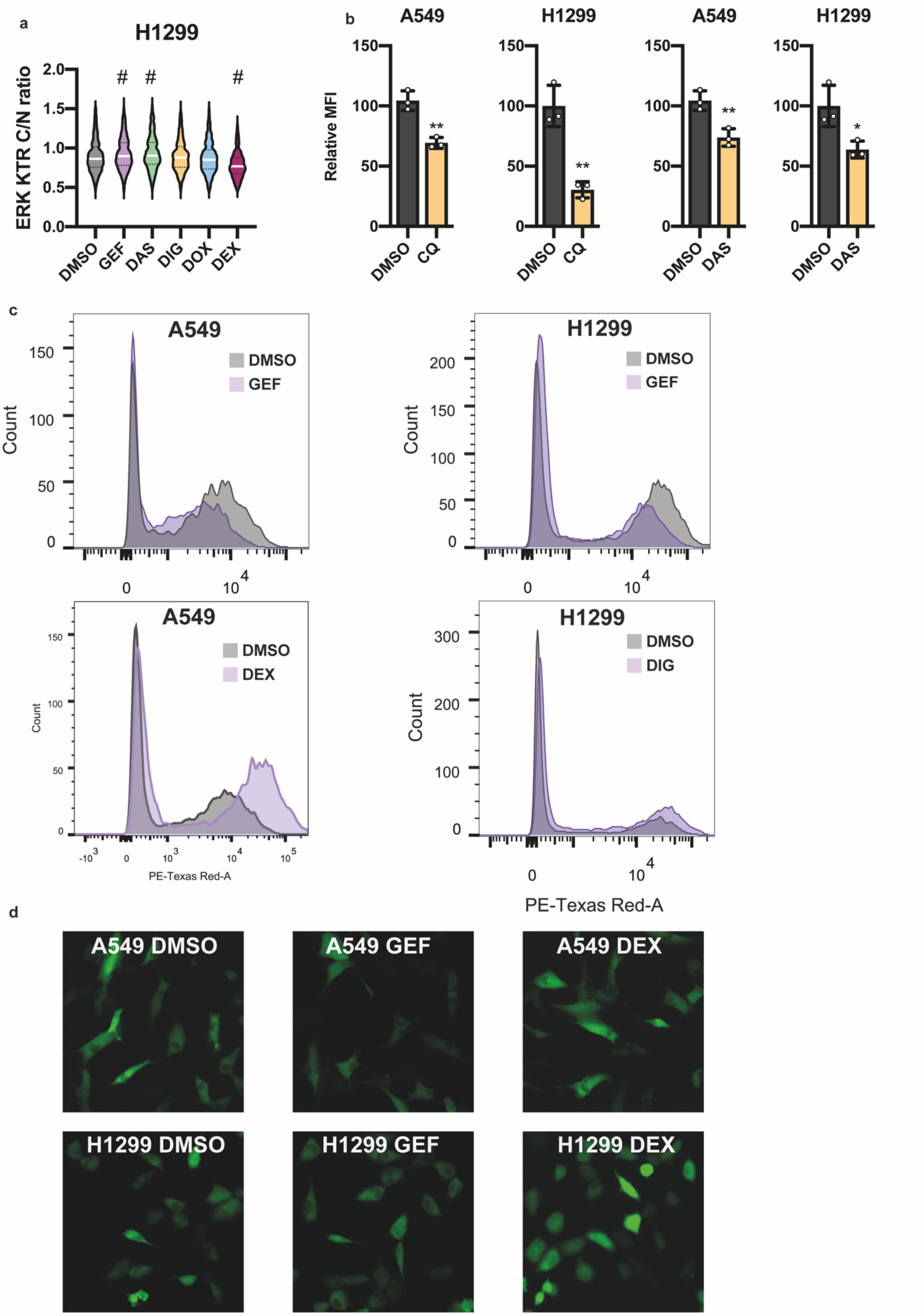

Figure S3 Drugs change the transduction efficacy of lung cells and the ERK activity. Related to Figure 3.

(a) Violin plots of normalized ERK activity in H1299 cells measured with ERK-KTR. H1299 cells with ERK-KTR cells were treated with drugs in FBS supplemented media for 6 hours.

(b) Relative MFI of the A549 and H1299 cells treated with 5 uM chloroquine (CQ) and 25 nM dasatinib (DAS). Error bars represent standard deviation from 3 independent biological replicates.

(c) Transduction efficacy of A549 (left panel) and H1299 (right panel) measured by flow cytometry in the presence of gefitinib (GEF), dexamethasone (DEX) or digoxin (DIG). DMSO treated cells were used as control.

(d) ERK-KTR sub-cellular distribution in A549 and H1299 cells treated with gefitinib (GEF) or dexamethasone (DEX) – images were obtained by fluorescent microscopy.

P-value for a was determined by unpaired t-test, * = p-value < 0.1, ** = p-value < 0.01. P-value for b was calculated by Mann-Whitney test, #- p-value< 0.001.

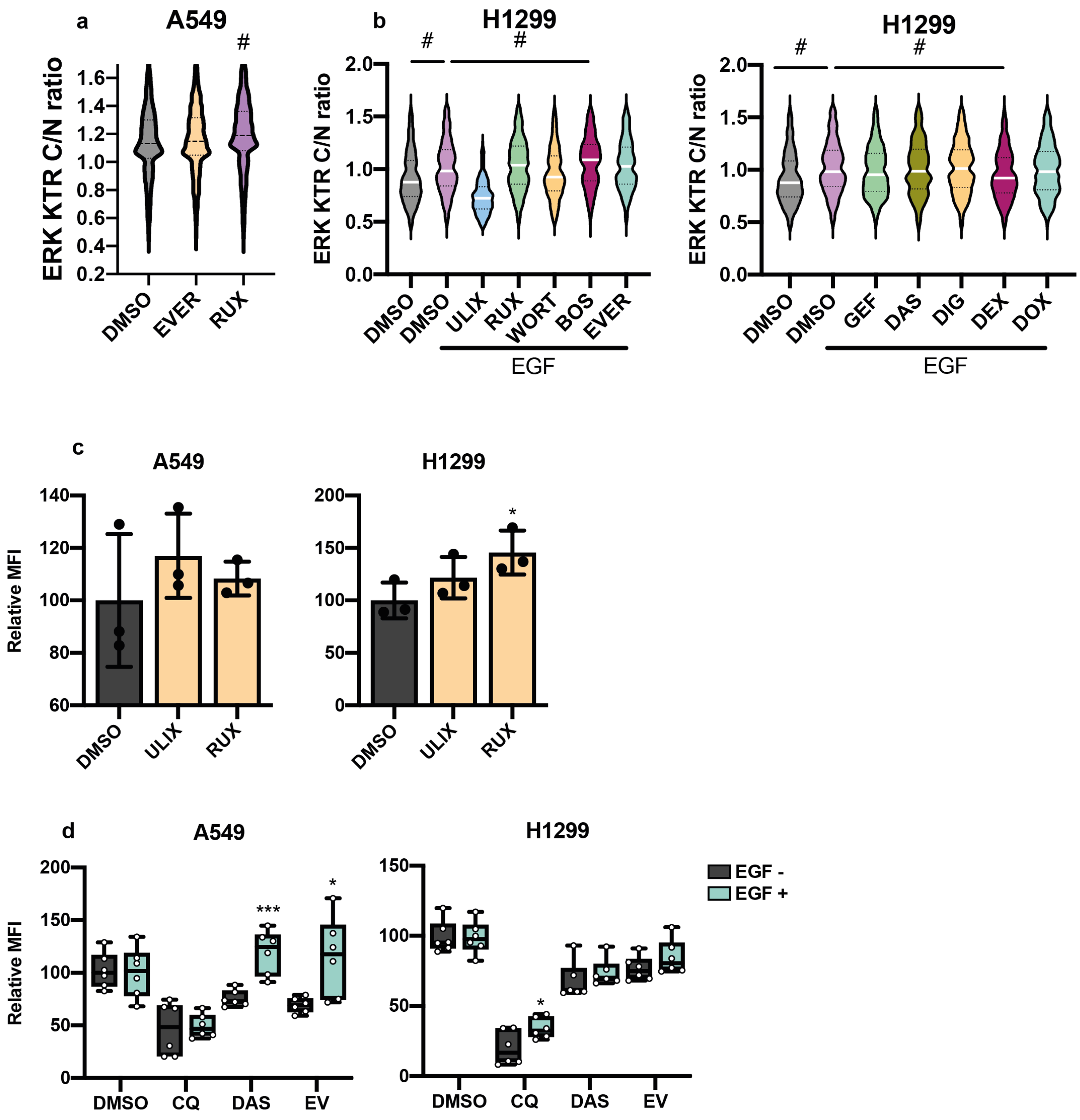

Figure S4 Drugs mediate EGF-driven ERK activation. Related to Figure 4.

(a) Violin plots of normalized ERK activity in A549 cells measured with ERK-KTR. A549 cells with ERK-KTR cells were treated with 100 nM everolimus (EVER) and 2.5 uM ruxolitinib (RUX) in FBS supplemented media for 6 hours.

(b) Violin plots of normalized ERK activity in H1299 cells measured with ERK-KTR. H1299 cells with ERK-KTR cells were treated with drugs (50 nM ulixertinib (ULIX), 10 uM ruxolitinib (RUX), 10 uM wortmannin (WORT), 1 uM) in media without FBS for 6 hours prior the addition of 100ng/uL recombinant EGF.

(c) Relative MFI of the A549 and H1299 cells treated with 50 nM ulixertinib (ULIX) and 2.5 uM ruxolitinib – A549 and 10 uM ruxolitinib – H1299 (RUX) prior the addition of lentiviral particles. Error bars represent standard deviation from 3 independent biological replicates. P-value was determined by unpaired t-test, * = p-value < 0.1.

(d) Relative MFI of the A549 and H1299 cells treated with 5 uM chloroquine (CQ), 25 nM dasatinib (DAS) and 100 nM everolimus (EVER) prior the addition of lentiviral particles. Data of 6 independent biological replicates represented as a bar plot with the max and min value indicated.

P-value for a, b was calculated by Mann-Whitney test, #- p-value< 0.001. P-value for c, d was determined by unpaired t-test, * = p-value < 0.1, ** = p-value < 0.01, *** = p-value < 0.001.

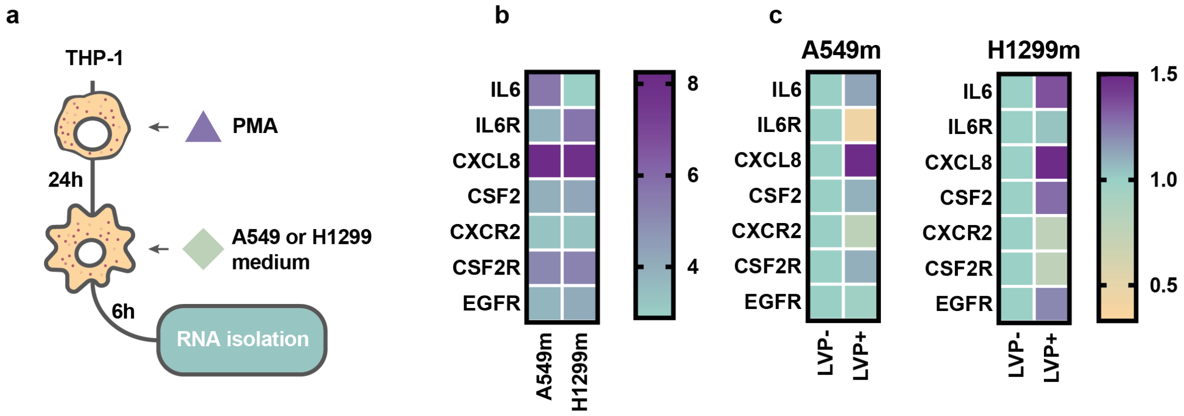

Figure S5. Lung cells conditioned medium changes the expression of cytokines in THP-1-derived macrophages. Related to Figure 5.

(a) Scheme of the experiment: THP-1 cells were differentiated into macrophages with PMA for 24 hours and incubated with the medium collected from A549 and H1299 cells.

(b) Heatmap with relative to GAPDH mRNA expression level of genes in THP-1 derived macrophages treated with A549 (A549m) or H1299 (H1299m) conditioned medium.

(c) Heatmap showing relative mRNA expression of genes in macrophages treated with medium collected from non-treated and LVP-infected lung cells normalized to DMSO treated control.

P-value was determined by unpaired t-test, * = p-value < 0.1, ** = p-value < 0.01, *** = p-value < 0.001.

Table S1 List of genes differentially expressed after SARS-CoV-2 infection of A549 and NHBE cells. Spearmen correlation coefficients of cyto-DEGs expression with ACE2 expression in lung tissue (correlations and p-values).

Table S2 List of cytokines and cytokine-related proteins elevated in COVID-19 patients and gene sets used for UMAP analysis.

Table S3 Pearson correlation matrixes of COVID-19 associated genes expression in IPF, healthy lung tissue, lung cancer and normal lung control tissue.

Table S4 (Cluster a). DEGs for cell lines in *cluster a* identified by UMAP based on expression of *cluster 1* genes. (Lung cancer group). DEGs for a distinct group of lung cancer samples identified by UMAP based on expression of *cluster 1* genes. GeneMania results for *cluster 1* genes. 14 genes were used for analysis (query) and 31 genes revealed by GeneMania (result).

Table S5 List of drugs identified in ChEMBL database, tested kinase inhibitors from DSigDB and drugs with possible inhibitory effects based on text mining data from DSigDB collection of computational drug signatures. For each inhibitor a list of possible anti-SARS-CoV-2 and anti-fibrotic targets is provided according to used database.

Table S6 (List 1) Vi-Fi scores for 121 drugs with known transcriptomic signature (from L1000 dataset). Table includes a list of alternative names, targets by which drugs were identified (combined from ChEMBL and DSigDB), their viral and fibrotic scores, main mechanisms of action and category to which each drug belong on figure 5C. **(List 2)** Combinational Vi-Fi scores for all 7260 combinations of 121 drugs with known transcriptomic signature. Viral and fibrotic score for individual drugs are also provided. **(List 3)** CMap score for 1915 drugs present in CMap database with non-zero fibrosis and viral scores.

Table S7 List of primers used in the study.

Table S8 List of publicly available datasets and their identifiers.
